# Ipsilateral lower limb motor performance and its association with gait after stroke

**DOI:** 10.1371/journal.pone.0297074

**Published:** 2024-02-02

**Authors:** Pei-Yun Lee, Chih-Hung Chen, Hui-Yu Tseng, Sang-I Lin

**Affiliations:** 1 Department of Physical Therapy, Medical College, National Cheng Kung University, Tainan, Taiwan; 2 Department of Neurology, Medical College, National Cheng Kung University, Tainan, Taiwan; 3 Department of Rehabilitation Medicine, Tainan Hospital, Ministry of Health and Welfare, Tainan, Taiwan; 4 Institute of Long-Term Care, MacKay Medical College, New Taipei, Taiwan; University of Verona: Universita degli Studi di Verona, ITALY

## Abstract

**Background and purpose:**

Motor deficits of the ipsilateral lower limb could occur after stroke and may be associated with walking performance. This study aimed to determine whether the accuracy and movement path of targeted movement in the ipsilateral lower limb would be impaired in the chronic stage of stroke and whether this impairment would contribution to gait.

**Methods:**

Twenty adults with chronic stroke and 20 age-matched controls went through Mini Mental Status Examination (MMSE), and a series of sensorimotor tests. The targeted movement tasks were to place the big toe ipsilateral to the lesion at an external visual target (EXT) or a proprioceptive target (PRO, contralateral big toe) with eyes open (EO) or closed (EC) in a seated position. A motion analysis system was used to obtain the data for the calculation of error distance, deviation from a straight path, and peak toe-height during the targeted movement tasks and gait velocity, step length, step width and step length symmetry of the lower limb ipsilateral to the brain lesion during walking.

**Results:**

The stroke group had significantly lower MMSE and poorer visual acuity on the ipsilateral side, but did not differ in age or other sensorimotor functions when compared to the controls. For the targeted movement performance, only the deviation in PRO-EC showed significant between-group differences (*p* = 0.02). Toe-height in both EXT-EO and in PRO-EO was a significant predictor of step length (R^2^ = 0.294, *p* = 0.026) and step length symmetry (R^2^ = 0.359, *p* = 0.014), respectively.

**Discussion and conclusions:**

The performance of ipsilateral lower limb targeted movement could be impaired after stroke and was associated with step length and its symmetry. The training of ipsilateral targeted movement with unseen proprioceptive target may be considered in stroke rehabilitation.

## Introduction

Stroke affects more than 80 million people globally and is the leading cause of disability among adults worldwide [1–3]. More than half of stroke survivors reported still having residual functional limitations in the chronic stage [4–6]. Sensory and motor impairments on the contralateral side of stroke are commonly accepted as the primary underlying cause of functional limitations. While this notion cannot be disputed, the function of the limbs ipsilateral to the lesion should not be overlooked, as it can help to compensate for the lost function and contribute to the maximization of functional ability [7–10].

Anatomical and imaging studies have shown that the descending pathways from the motor cortex, although predominantly cross to the contralateral side, there remain some ipsilateral connections [11]. Stroke may affect the origin of these uncrossed connections and lead to ipsilateral motor deficits. Imaging studies have also shown bilateral cortical activation during unilateral hand movements, suggesting bilateral hemispheric control of movement [12, 13]. Thus, brain lesion on one side could affect motor performance bilaterally. When taken together, these notions suggest the possibility of ipsilateral motor deficits after a stroke.

For the ipsilateral upper limb, motor deficits could be observed soon after stroke [14–16]. In the chronic stage of stroke, ipsilateral upper limb motor deficits continue to exist, including lower muscle strength [17, 18], slower movement time [8, 17, 19–21], larger aiming error [17, 22, 23], and poorer dexterity [18] and force regulation [24], compared to non-impaired age-matched controls. Some of these ipsilateral deficits have been found to be related to the performance of daily activities [18, 25, 26].

For the lower limb motor deficits ipsilateral to the lesion, the information is scarce. Immediately after stroke, the ipsilateral quadriceps showed smaller peak isometric torque and poorer force regulation [27]. The ability of ipsilateral foot tracking was also found to be impaired, i.e. slower and less accurate, in the subacute stage of stroke [28]. In the chronic stage, when performing fast repeated target tapping with the ipsilateral foot, stroke patients were found to require longer time between landing and liftoff [20]. In both previous studies of ipsilateral lower limb movement control, external visual targets were used for the study of tracking movements. However, in daily living, the foot ipsilateral to the lesion may need to make discrete movements or move to a location in relation to the contralateral foot, i.e. proprioceptive target. What is more, it is unclear if ipsilateral motor deficits would be associated with declined walking performance after stroke.

The purposes of this study were to determine whether the accuracy and movement path of targeted movement in the ipsilateral lower limb would be impaired in the chronic stage of stroke and whether this impairment would be related to gait. It was hypothesized that for the stroke group, their performance of ipsilateral targeted movement, including accuracy and movement path, would differ from the controls, and these performances would be associated with gait. Specifically, this study used external and proprioceptive targets to simulate the need of daily activities. The information could improve the understanding of motor deficits after stroke and provide information for the planning of stroke rehabilitation in the chronic stage.

## Methods

### Study participants

Persons with stroke who visited outpatient clinics at the neurology department of a medical center and the rehabilitation department of a local hospital during the experimental period were screened for eligibility and invited to participate. The inclusion criteria included having a unilateral first time **cortical/subcortical** stroke at least 6 months ago, medically stable and able to understand instructions and follow experimental commands. The exclusion criteria included full or near full recovery of the lower limb motor function (Brunnstrom motor recovery stage VI or better) or other neuromuscular or musculoskeletal conditions that would interfere with the lower limb movements or walking. Forty-seven stroke patients were contacted. Among them, 21 patients were excluded because of not meeting the inclusion criteria or refusing to participate in the study. Another six patients were unable to complete the experimental procedure because of fatigue. A group of age-matched controls (CON) from nearby communities was also recruited. The Institutional Review Board of the National Cheng Kung University Hospital approved this study. All the research methods were performed in accordance with the provisions of the Declaration of Helsinki (as revised in Tokyo 2004). All the participants provided written informed consents. The corresponding author has access to information that could identify individual participants during or after data collection. After enrollment, the participants received sample characterization, targeted movement, and walking tests sequentially.

### Sample characterization

For the characterization of the study participants, a series of sensorimotor function tests were conducted. The modified Traditional Chinese version of the Mini Mental State Examination (MMSE, maximal score = 33) has been found to have high reliability and was used to assess cognitive function [29]. This version added three items, subtraction (7 minus 3), addition (2 plus 4), and writing down one’s name, to account for the impact of low education level. Visual acuity was tested with a standard printed Snellen eye chart 6 m away. The Snellen eye chart is widely used for clinical visual acuity assessment due to its ease of use, even though its reliability has been demonstrated to be poor [30]. The Fugl-Meyer lower extremity motor scale (FMLE-motor**, range 0–34**) has been shown to have high reliability [31] and was used to measure the motor function of the lower limb contralateral to the stroke. **A score of 21 or higher has been shown to indicate a high level of mobility function in persons with chronic stroke [32].** The scale included the test of reflex activity of the knee flexors and extensors, volitional movement within synergies, volitional movement mixing synergies, and volitional movement without synergy, and coordination (heel-to-knee cap in supine). Because the majority of the stroke participants showed involuntary associated movements in the contralateral lower limb, the leg muscle strength was not directly measured. Grip strength, a measure which has been shown to be highly correlated with the strength and motor function of the lower limbs [33, 34], was measured using a handgrip dynamometer. Plantar cutaneous sensitivity was examined at the plantar side of the first metatarsal head using the Semmes-Weinstein monofilaments (Patterson Company, IL, USA). Briefly, the monofilament fiber was applied at a 90° angle to the skin, and the participant indicated if the fiber was felt or not. The smallest filament that the participant was able to perceive indicated the threshold for touch-pressure. The test has been found to have high reliability [35].

A limb matching task, that is moving the ipsilateral limb (less affected side) to match the position of the contralateral limb, is one of the frequently used tests of joint position sense for persons with stroke [3, 36, 37] and was adopted in this study to measure the knee and ankle joint position sense. Participants closed their eyes and sat in a customized, height-adjustable chair with fully supported thighs, and feet freely dangling above the ground. To test the knee joint position sense, the experimenter held the bilateral malleoli to move the nondominant (for CON) or contralateral (for **STROKE**) lower leg approximately 15° into flexion or extension, stopped, and then instructed the participant to match the knee joint angle by moving the other lower leg without moving the rest of the body. To test the ankle joint position sense, the same initial position was adopted except that both heels were supported by a stool. The experimenter first held the first and fifth metatarsal heads to move the nondominant (for CON) or contralateral (for **STROKE**) ankle joint into plantar- or dorsi-flexion, stopped, then instructed the participant to match the ankle joint angle by moving the other foot without moving the rest of the body. Each task was repeated twice. The differences in the joint angle of the two sides were used to represent joint position sense.

### Targeted movement tests

Participants sat in a customized height-adjustable chair with the trunk erect (no back support), buttocks near the front edge of the seat, knees slightly bent and feet flat on the ground. This starting position was designed to simulate upright standing position in which most of the lower limb functional movement would occur, but with the body weight supported by the chair to minimize the need for balance control. Participants were asked to move the dominant (for CON) or ipsilateral (for **STROKE**) foot to a target on the floor that was covered by black cloths. In daily life, the foot may need to move to a particular location in relation to an external target, such as a slipper, or a proprioceptive target, such as the other foot, either with or without online visual cues. Thus, two types of targets were used, external and proprioceptive, and for each target, the task was performed twice with and without vision. The target would be positioned at a distance approximately one third of the foot-length away from the moving foot’s big toe. The participants practiced each condition once before data acquisition. The tests were conducted using either the dominant (for CON) or ipsilateral (for **STROKE**) leg. The test sequence for the proprioceptive and external target conditions was randomized to reduce the potential impact of fatigue or learning.

#### External target

A bright-colored arrowhead (target) was placed on the ground, approximately one third of the foot-length away from the big toe, at a distance easily within reach by the moving foot (dominant side for CON and ipsilateral side for **STROKE**). Participants were allowed 3s to look at the target and memorize its location. Afterwards, the target was removed and the participants were instructed to move the big toe to the memorized target location. In the eyes open condition (EXT-EO), the eyes remained open for the entire test. In the eyes closed condition, participants closed their eyes before moving the foot (EXT-EC).

#### Proprioceptive target

This task required the participant to place the dominant (for CON) or ipsilateral (for **STROKE**) big toe at the location previously occupied by the big toe (target) of the nondominant/contralateral foot. The starting position was identical to that of the external target condition. The target foot was moved passively to a random location within reach of both feet (approximately one-third foot-length in front and within 30° range of its initial location). Participants were allowed 3s to memorize the target location. Afterwards, the feet were first lifted off the floor and then the chair was rotated approximately 30° around the vertical axis toward the side of the target foot after the participants opened their eyes. This condition was designed to simulate a situation where the foot ipsilateral to the lesion was to move to a position in relation to the contralateral foot after the position of the body has been changed in the environment, similar to walking. In the eyes open condition (PRO-EO), the eyes remained open throughout the test. In the eyes closed condition (PRO-EC), participants closed their eyes when the contralateral foot was being passively moved, and then opened the eyes after the chair rotation.

### Walking test

Participants were instructed to walk at their preferred speed on an obstacle-free walkway for 8 meters and used their usual walking devices as needed. The test was performed once.

### Data reduction and statistical analysis

SIMI MOTION® (SIMI Reality Motion Systems GmbH, Unterschleissheim, Germany) with an eight-camera 3 dimensional motion analysis system was used to record the lower limb kinematics with a sampling rate of 100 Hz for joint position sense, targeted movement and walking tests. For the joint position sense assessment and walking test, reflective markers were placed at the midpoint between the two posterior superior iliac spines, and bilateral anterior superior iliac spines, greater trochanters, medial and lateral epicondyles of the knees, medial and lateral malleolus, second metatarsal heads, and heels in order to calculate the knee and ankle joint angle. Two reflective markers were placed at the nails of both big toes to indicate their positions during the targeted movements and an additional marker was used to indicate the location of the arrow for the external target conditions.

Computer algorithms written in MATLAB (Version R2013a, The Math Works Inc. MA, USA) were used for data reduction. For the joint position sense assessment, the hip, knee and ankle joint centres were calculated according to the joint kinematic methods proposed by the International Society of Biomechanics [38]. The hip and knee joint centre formed the thigh segment, the knee and ankle joint centre formed the shank segment, and the heel and second metatarsal head formed the foot segment. The knee joint angle was defined as the angle between the thigh and shank segments. The ankle joint angle was defined as the angle between the shank and foot segments. For the external target and exproprioception tests, the position data of the reflective markers were used to calculate the errors and deviations.

**Fig 1:**
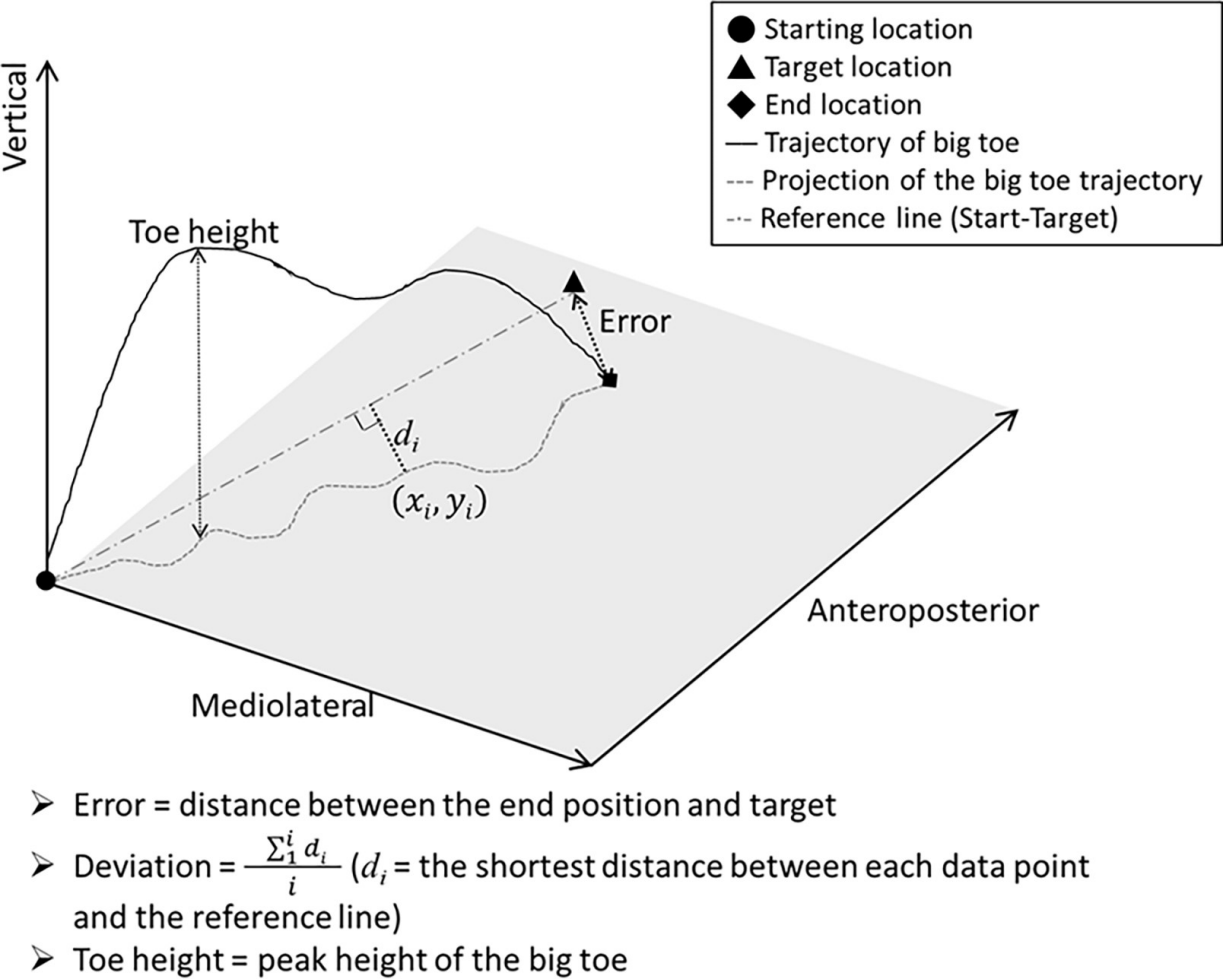
Definitions of targeted movement task parameters (error, deviation, toe-height). The continuous line shows the trajectory of the big toe of the moving leg.

The parameters of interest for the targeted movement included the error, deviation, and peak height of the big toe on the side ipsilateral to the lesion (Fig 1). The distance between the final position of the big toe and the target was used to represent error. The mean distance between the big toe (of each data point) and the line between the starting position and the target was used to represent the deviation. Toe-height was the highest point of the big toe trajectory.

For walking performance, the means of two consecutive strides were used for data analysis. Because the parameters for targeted movement primarily concerned spatial characteristics, the gait parameters of interest thus focused on step length and its symmetry index, and step width. Gait velocity was also investigated to represent overall gait performance. Symmetry index (SI) was calculated based on the equation below.

$$SI=\left|1-\frac{ipsilateral}{contralateral}\right|$$

where ipsilateral = the side ipsilateral to the brain lesion

For the basic information and physical function, independent *t*, Mann-Whitney U, and *chi* square tests were used for between group comparisons for continuous, rank ordinal and categorical variables, respectively. For the targeted movement and gait parameters, because most of them were not normally distributed, the Mann-Whitney U tests were used for between-group comparisons. To assess the potential explanation of variance in the gait performance of the ipsilateral lower limb for the stroke group by the targeted movement performance, a stepwise regression analysis was performed. In this analysis, the gait parameter was treated as the dependent variable, while its corresponding significantly correlated targeted movement parameter was considered as the independent variable. Furthermore, considering that the gait characteristics of the limb ipsilateral to the lesion might be associated with the motor function of the contralateral limb, the FMLE-motor scale would also be included as an independent variable in the regression analysis, if it demonstrated a significant correlation with gait performance. Kendall’s tau’b correlation analysis was used to determine the correlations between the gait parameters, FMLE-motor and targeted movement parameters. The significance level was set at 0.05.

## Results

**Fig 2:**
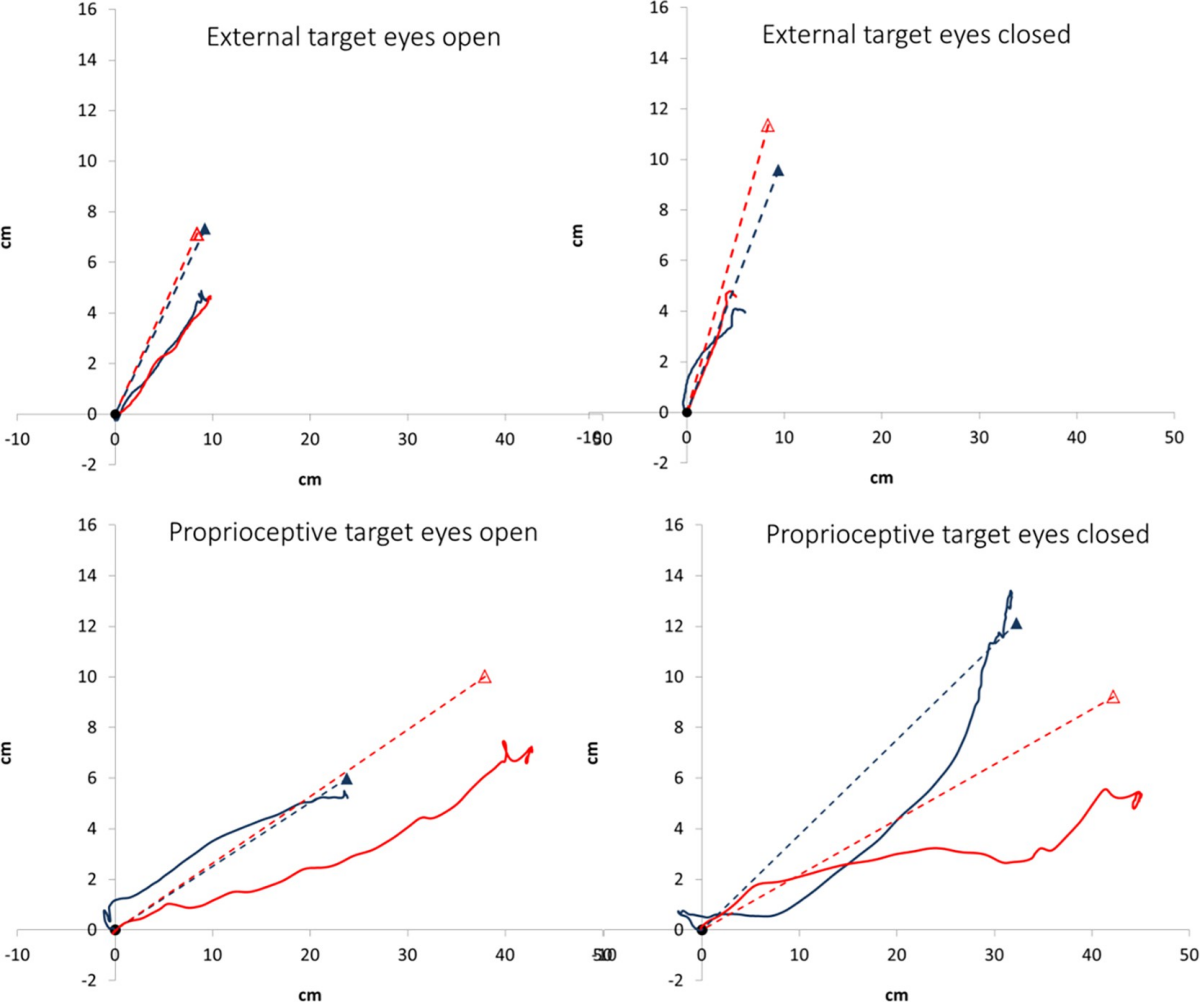
The big toe trajectory of the ipsilateral leg targeted movement tasks of a representative control (blue lines) and stroke (red lines) participant. Circle: starting position; triangle: target location; continuous line: trajectory of the big toe; dotted line: direct line between the starting position and target in the sagittal plane.

There were 20 participants in each group. Compared to CON, **STROKE** had significantly lower MMSE and poorer ipsilateral side (comparing to the dominant side of CON) visual acuity. Comparing to the dominant side of CON (Table 1), the ipsilateral side of **stroke** had significantly poorer visual acuity, but not grip strength or plantar sensitivity. Comparing to the non-dominant side of CON (Table 1), the contralateral side of **stroke** had significantly smaller grip strength, but not visual acuity or plantar sensitivity. Regarding the targeted movement performance, it was observed that only the deviation in PRO-EC exhibited significant differences between the two groups. Specifically, **STROKE** displayed a greater deviation in PRO-EC compared to CON. (Table 1). Fig 2 displays examples of targeted movement trajectories under various conditions. The gait parameters were all significantly different between the two groups (Table 1).

**Table 1:** Basic characteristics, targeted movement performance and gait. Ipsilateral and contralateral side refers to the side Ipsilateral and contralateral to the stroke, respectively.

	Control	Stroke	*p*
Age (years)	61.9±8.0	61.8±8.3	0.85
Mini Mental Status Examination	31.9±1.0	29.6±3.7	0.005*
Stroke			
Side (right/left)		10/10	
Duration (month)		33.8±30.4	
Fugl-Meyer lower limb motor scale		19.4±5.1	
**≥21/<21**^**#**^		**11/9**	
Dominant/ipsilateral side			
Visual acuity (decimal)	0.70±0.33	0.47±0.24	0.019*
Plantar sensitivity big toe (dB)	4.00±0.17	4.08±0.59	0.221
Grip (% body weight)	38±12	37±10	0.642
Non-dominant/contralateral side			
Visual acuity (decimal)	0.63±0.31	0.58±0.27	0.708
Plantar sensitivity big toe (dB)	4.01±0.19	4.21±0.46	0.076
Grip (% body weight)	35±10	16±17	0.002*
Joint position error (degree)			
Knee	4.90±4.24	5.28±2.64	0.737
Ankle	6.49±2.75	7.32±5.03	0.534
Targeted movement performance			
External target			
Error (cm)			
Eyes closed	3.05±1.14	3.29±1.21	0.512
Eyes open	2.05±0.60	2.02±0.51	0.904
Deviation (cm/data point x 100)			
Eyes open	0.72±0.28	0.65±0.21	0.718
Eyes closed	1.15±0.45	1.43±0.74	0.301
Toe-height (cm)			
Eyes open	4.62±1.82	5.53±1.92	0.105
Eyes closed	4.40±1.55	5.24±2.13	0.174
Proprioceptive target			
Error (cm)			
Eyes open	2.59±1.65	3.20±2.46	0.383
Eyes closed	4.86±2.31	7.21±4.40	0.091
Deviation (cm/data point x 100)			
Eyes open	2.08±1.26	2.35±1.42	0.565
Eyes closed	2.37±0.72	3.74±2.05	0.02*
Toe-height (cm)			
Eyes open	6.72±2.87	7.55±2.87	0.231
Eyes closed	6.90±2.28	7.57±2.60	0.398
Gait parameters (ipsilateral side)			
Gait velocity (m/s)	1.17±0.18	0.49±0.35	<0.001*
Step length (m)	0.61±0.09	0.33±0.19	<0.001*
Step width (m)	0.10±0.03	0.14±0.04	0.001*
Symmetry index of step length	0.15±0.09	2.07±5.20	0.039*

*statistically significant

^**#**^ **A Fugl-Meyer lower limb motor scale ≥21 indicated a high level of mobility function.**

Table 2 shows the results of correlation and regression analysis for **STROKE**. Gait velocity and step width were not significantly correlated with any of the targeted movement parameters, and thus regression analysis was not conducted. The ipsilateral step length was significantly correlated with deviation and toe-height in EXT-EO, and FMLE-motor. The variables EXT-EO toe-height and FMLE-motor explained 29.4% and 17% of the variance in ipsilateral step length, respectively. The symmetry index of step length was significantly correlated with toe-height in EXT-EO and PRO-EO, and FMLE-motor, with PRO-EO toe-height explaining 32.3% of the variance.

**Table 2:** Results of correlation and regression analysis.

Dependent factor	Independent factors (correlation coefficient)^#^	*R*^2^ change	Beta coefficient	95% CI	*p*
Gait velocity					
	None				
Step length					
	EXT-EO deviation (-0.490)	NI			
	EXT-EO toe-height (-0.412)	0.294	-0.474	-2.751~-0.206	0.026*
	FMLE-motor (0.485)	0.170	0.418	0.033~2.920	0.046*
Step width					
	None				
Symmetry index of step length					
	EXT-EO toe-height (0.386)	NI			
	PRO-EO toe-height (0.359)	0.323	0.569	0.565~4.284	0.014*
	FMLE-motor (0.364)	NI			

^#^ factors significantly correlated with the dependent factor in correlation analysis

CI: confidence interval

NI: not included in the regression model

FMLE-motor: Fugl-Meyer lower limb motor scale

## Discussions

After stroke, the function of the ipsilateral limbs could be important. This study focused on the ipsilateral lower limb targeted movement of stroke participants and found that, compared to the controls, the movement path was less straight when the target was the un-seen contralateral foot. Furthermore, some of the ipsilateral targeted movement parameters could explain more than a quarter of the variance in step length and its symmetry during walking for the stroke group. These findings suggest that ipsilateral lower limb function could be impaired after stroke and could affect walking performance.

To move a body end point (such as hand or foot) to an intended location in the environment, several functions are needed, including sensory information about the target with respect to the body and about the initial position of the body, transformation of this information into motor commands, and sending out motor commends for movement execution [39–41]. In this study, the between-group difference in the targeted movement were not significant when the target was clearly seen, i.e. in EXT-EO and EC and PRO-EO. Therefore it seemed that for the ipsilateral lower limb in persons with stroke, these functions were not likely to be impaired to an extent that would affect the accuracy or movement path of targeted movement performance.

To reach a target without any temporal or spatial constraints, the most efficient movement path would be a straight line from the starting point to the target, and the hand tends to move fairly straight when the target location is known [42]. Studies have shown that when there is uncertainty about the target, greater deviations in the hand path would occur in monkeys, while humans would tend to exhibit greater path length in reaching movements [43–45]. Insufficient sensory inputs or errors in sensory estimates of the target could result in target uncertainty and possibly lead to deviations in movement path [43, 46]. In the proprioceptive target conditions in this study, the location of a body segment in the environment was the target. The perception of the orientation of the body in extra-personal space relies on visual, proprioceptive and exteroceptive inputs, however these signals alone are insufficient to provide this information [47–53]. It has been proposed that these signals when mapped onto a body representation could provide information about the body in extra-personal space [47, 52–54]. The awareness of the limb orientation in the environment can also enable the planning of motor commands to move the limb directly toward a target outside the body [55].

In PRO-EC, there were no visual cues for the estimation of the target location and it was found that the movement path of the stroke group had significantly greater deviation, compared to the controls. Moreover, the absence of a significant between-group difference in PRO-EO also suggested that the between-group differences observed in PRO-EC could mainly be attributed to the differences in visual cues. It appeared that the stroke group was affected to a greater extent by a lack of visual cues about the target. These findings also implied that the ability to estimate the location of the contralateral foot in the environment without visual cues might be affected after stroke.

**Previous studies have reported that possibly due to deficits in motor control mechanisms, such as movement planning and organization, stroke may lead to declines in the performance of ipsilateral upper limb aiming movement [8, 20, 56]. For the lower limb, in addition to sensorimotor function of the contralateral lower limb, muscle strength in the ipsilateral lower limb have also been shown to exhibit associations with gait patterns after unilateral stroke previously [57–60]. This study included persons with chronic stroke with different mobility function levels and** was the first to report that ipsilateral lower limb targeted movement performance was also associated with gait after stroke. **The sub-tasks involved in gait adaptation primarily include taking a step to properly place the foot in the environment and at the same time maintaining forward propulsion force and balance in healthy adults and persons with stroke [61, 62]. Targeted movement is a fundamental ability in proper foot placement and could thus be associated with gait.** Specifically, in the external and proprioceptive target eyes open conditions, greater toe-height predicted shorter step length and poorer step length symmetry. For the stroke group, while the motor function (FMLE-motor) of the contralateral lower limb was found to be a significant factor influencing step length, its impact was relatively minor compared to the performance of targeted movement in the proprioceptive target eyes closed condition (PRO-EC).

It should also be noted that in this study, the big toe and the target were placed on the ground and therefore lifting the foot off the floor was not necessary. In spite of this, all the participants lifted the foot off the floor while moving toward the target. Intuitively, this behavioral strategy was used to avoid foot-ground contact. During the swing phase of walking, the swing foot also needs to avoid floor contact. While walking under more challenging conditions, such as vision restrictions or floor irregularity, attention would be consciously diverted toward the control of the lower limbs. This could lead to increased minimal distance between the swing foot and the floor [63–65]. In the eyes open condition in this study, continuous online visual cues about the distance between the foot and the floor were available and could be consciously used for the control of the movement. Therefore, lifting the foot higher off the floor, resulting in greater toe-height, could serve as an indicator of heightened attentiveness to the control of the swing foot. For stroke participants, this shift in attention could possibly mean taking smaller steps or allocating less attention to maintaining gait symmetry in order to minimize foot-floor contact. Further studies are needed to verify these notions.

This study is limited in several ways. The psychometric properties of the targeted movement performance measurements were not tested and could limit the reliability of the data. The mechanisms underlying the association between targeted movement performance and gait were not investigated. The contributions of the sensorimotor function of the lower limbs contralateral to the stroke were not included in the regression analysis due to sample size limitations. Future studies are needed to better understand these issues.

In conclusion, persons with stroke did not differ from healthy controls in performing ipsilateral lower limb targeted movement when the target location was visible prior to initiating the movement. When the target was the contralateral foot and not visible, the motion of the ipsilateral leg showed significantly greater deviation, suggesting impaired ability to estimate the location of the contralateral foot within the environment without visual cues after stroke. The performance of ipsilateral targeted movement within the stroke group accounted for over a quarter of the variation in both step length and its symmetry. These findings together **implied that the training of ipsilateral targeted movement may be helpful in improving walking performance for people with chronic stroke. However, future studies are needed to examine the possible effects.**
